# Treatment outcomes and factors affecting time-to-recovery from severe acute malnutrition in 6–59 months old children admitted to a stabilization center in Southern Ethiopia: A retrospective cohort study

**DOI:** 10.1186/s13052-019-0642-x

**Published:** 2019-04-11

**Authors:** Anteneh Fikrie, Akalewold Alemayehu, Samson Gebremedhin

**Affiliations:** 1 Community Service and Research Directorate, Pharma College Hawassa Campus, P.O.B: 67, Hawassa, Ethiopia; 2 School of Public Health, College of Medicine and Health Sciences, Hawassa University, Hawassa, Ethiopia; 3 St. Paul Hospital Millennium medical College, Addis Ababa, Ethiopia

**Keywords:** Complicated SAM, Survival status, Recovery time, Predictors, Ethiopia

## Abstract

**Background:**

Despite improving access to Severe Acute Malnutrition (SAM) management, information on the quality of the service, as measured by timely recovery, is scare. This study is designed to assess treatment outcomes and factors affecting time-to-recovery from SAM in children 6–59 months admitted to a stabilizing center in Hawassa University Comprehensive Specialized Hospital (HU-CSH), Southern Ethiopia.

**Methods:**

Institutional-based retrospective cohort study was conducted on 420 randomly selected children aged 6–59 months. The children were managed at the hospital from July, 2015 to June, 2017. Pre-tested structured questionnaire was used to extract data from medical records. Data were analyzed using Kaplan Meir (KM) curve, Log rank test and Cox-Proportional hazards model. The outputs of the bivariable and multivariable Cox model are presented using Adjusted Hazard Ratio (AHR) with the respective 95% Confidence Intervals (CIs).

**Results:**

After a maximum of 59 days treatment 69.3% of the children recovered and 10.8% died. The mean (±SD) weight gain rates was 12.7 (±8.9) g/kg/days. The overall incidence density rate of recovery was 3.8 per 100 person-days. The overall median (IQR) time of recovery was 17(10, 24) days. F-100 intake (AHR = 0.502, 95%, CI: 0.29–0.86), Tuberculosis infection (AHR = 1.38, 95% CI: 1.00–1.91) and provision of special medication (IV fluid, IV antibiotic and blood transfusion) (AHR = 0.72, 95% CI: 0.52–0.99) at admission were found to be significant predictors of time-to-recovery from SAM.

**Conclusion:**

The overall recovery from complicated SAM children admitted at HU-CSH after a maximum of 59 days treatment was low (69.4%) and a very high proportion of children (10.8%) end up in death. Therefore, HU-CSH should give special focus for those children present with medical comorbidities during admission.

## Background

Severe Acute Malnutrition (SAM) is defined as a very low weight for height/length (below − 3-z scores of the median World Health Organization (WHO) growth reference, or below 70% of the median of National Centre for Health Statistics reference) and by the presence of nutritional edema [1]. According the the national protocol of Ethipoia, SAM is defined as children 6–59 months with < − 3 z-scores, and/or MUAC < 11.5 cm, and/or bilateral pitting nutritional oedema [2].

SAM is the most extreme and visible form of under nutrition and looms the survival of children under-five years of age [3]. Of estimated 555 million global children under five years of age, nearly, 19 million are severely wasted. More than 90% of them live in the developing countries, specfically in Sub-Saharan Africa and South East Asia [3]. According to the recent Ethiopian Demographic and Health survey (EDHS) report, wasting and severe wasting is estimated to be 10 and 3% among under five children respectively [4]. Likewise, in Southern Nations, Nationalities and Peoples’ Region (SNNPR), 6% of children were wasted and 1.7% were severely wasted [4].

Acute malnutrition, or wasting, exclusively is an attributable cause of 12.6% of the 6.9 million deaths among children under 5 years old [5]. Furthermore, specially in sub-Saharan and South-East Asian countries, it is responsible for nearly one million deaths each year by increasing susceptibility to death from severe infection [3]. Mortality rate due to SAM, among Ethiopian children treated in Stabilizing Center (SC) was observed as high as 29% [6].

A study indicated severely wasted children have eleven times higher risk of death as compared to their counterparts [7]. Similarly another evidence suggests that children suffering from SAM have a 5–20 times greater risk of death than well-nourished children [8]. In addition to increasing the risk of death and disease, SAM also leads to growth retardation and impaired psychosocial and cognitive development [9]. SAM has economic ripple effects that can jeopardize development. Globally, the direct cost of SAM is estimated at $20 to $30 billion per year [10].

According to the community management of acute malnutrition (CMAM) guideline of Ethiopia, SAM is managed either in outpatient or impatient basis. Patients with good appetites and no major medical complications or have + and ++ oedema will be linked to the out-patient treatment program. Whereas patients with failed appetite, and/or with a major medical complication and children with +++ oedema are initially admitted to an in-patient facility for Phase 1 treatment [2].

Despite being in a good track of progress in reduction of under-five mortality rate, Ethiopia is still suffers from SAM which has been remains remarkably huge and at the same time its prevalence has not been significantly reduced during the past three decades [4]. A hospital based study in Ethiopia revealed an alarming low cure and high death rate of 46 and 29%, respectively [6]. Such high mortality in inpatient units has been attributed to either co-morbidities such as HIV infection and diarrheaor to poor adherence to the existing therapeutic guidelinesb [11]. In Ethiopia 82% of all cases of child malnutrition and its related pathologies are not appropriately treated or left untreated [12].

Therefore, the intention of this study is toassess treatment outcome and factors affecting recovery time from SAM among 6–59 month’s old children in HUSCH.

## Methods

### Study design and study area

Institution-based retrospective cohort study design based on secondary records of children aged 6–59 months with SAM was conducted from January 1 to January 10, 2018 at HU-CSH stabilization center, Southern Ethiopia. The hospital is located in Hawassa city, the capital of SNNPRS. It is one of the teaching and specialized and comprehensive hospital in the Southern part of Ethiopia. It has over 400 beds; out of which 40 beds are in the pediatric ward and half of pediatrics ward beds are reserved for treatment of SAM children. Pediatric ward has a corner for treatment of SAM patients with the phased approach of the of the WHO protocol [1]; further, SAM patients admission, treatment and discharges is made according to the national protocol for SAM management [2]. All admissions are examined by rotating resident physicians, internship medical doctors, and nurses, who are responsible in fill up the therapeutic multi-chart for SAM management.

The patient would be screened for inpatient or outpatient treatment based on the Ethiopian standard. First, the patient is identified in the community by MUAC and oedema. The severely ill are “fast tracked” to treatment by the person doing the screening. Then appetite test is performed while waiting to see the health care provider who looks for the presence of medical complications. Health care provider discusses with the caregiver and decides upon the appropriate treatment options. Those that need in-patient treatment are referred for admission to inpatient care/SC; those that can be treated as out-patients are referred to the outpatient therapeutic program (OTP) site nearest to their home [2].

### Sample size and sampling procedure

Sample size for assessing treatment outcomes was calculated using single population proportion formula. The specifications made during the computation were: expcted recovery rate of 46% [6], 95% confidence level, 5% margin of error and 10% compensation for possible missing values. The ultimate sample size was calcuated as 420. Inorder to identify factors associated with SAM, the adequacy of the sample size was examined using post-hoc power calcuation.

Eligible cases were selected from the exisiting medical records using simple random sampling technique. The sampling frame was developed for all records of SAM patients aged 6–59 months of age admitted in HUCSH stabilization center form July, 2015 to June, 2017 based on their unique SAM identification number.

### Data collection procedures and quality

Five trained clinical nurses working at stabilizing center and a Public health officer were participated in the data extraction and supervision, respectively. Both the data collectors and supervisor were trained for two-days on how to extract data, what to be extracted and to make to internalize the context of each question in the checklist. A pretested structured extraction form was used to collect data.

Data like socio-demographic characteristics (age, Sex of the child, Place of residence, Breastfeeding), Medical co-morbidity(pneumonia, HIV sero status, Diarrhea, Dehydration status, Hemoglobin level, malaria),routine medication and treatment (IV fluid intake, IV antibiotic treatment, blood transfusion, folic acid and Vitamin A supplementation, F 100 and F 75 intake), Anthropometry and type of malnutrition (WFH, MUAC, non-edematous and edematous), and treatment outcome were extracted from SAM children’s records.

According to the Ethiopian national protocol, SAM treatment outcome is classified into: recovery, defaulted, transfered out, death, non-responded and refered. Non-responder is a patient that has not reached the discharge criteria after 40 days in the inpatient care. Length of stay is the number of days the child stayed in the hospital from admission until the child develop event of interest (cure) or censored (defaulted, died, transfer out, non-responded and medically transfered).Length of stay was computed for recovered children one by one using the difference between the date of admission to SC and the date of discharge [2].

### Data processing and analysis

The data was thoroughly cleaned, coded and then entered in to EPINFO version 3.5.3 and exported to the Statistical Package for Social Science (SPSS) version 20 for analysis. Descriptive analysis was ran to assess missing values and presence of outliers. The Kolmogrov Smirnov test of normality was used to check the normality of distributions for continuous variables. The dependent outcome variable was time to recovery (the time it takes for severely malnourished children to attain nutritional recovery). The treatment outcome was dichotomized into recovered or censored. All treatment outcomes other than recovered were regarded as censored. Multicollinearity test were carried to see the correlation between the independent variables and no correlation between independent variables was witnessed (Variance inflation factor < 10).

Data were described using frequency distribution and measures of central tendency and dispersion. Kaplan- Maier Curve and Long rank test were used to estimate cumulative survival probability and to compare survival status probablity across different groups. Bivariable and multivariable Cox proportional-hazard regression were used to identify predictors of time-to-recovery. Variables with *p*-value less than 0.25 during bivariate analysis were considered as a candidate for multivariate analysis. The assumption of proportional hazard was graphically evaluated by log-minus-log survival curve. Adjusted hazard ratio (AOR) with 95% confidence interval (CI) were used to present the output of the analysis.

## Results

### Characteristics of the children at admission

From a total of 420 randomly selected records of children with SAM, the data of 381 with complete medical records were extracted with a retrieval rate of 91.2%. Majority 265 (69.6%) of the children came from rural areas. The mean (± SD) age of the children was 22.4(±15.8) months and the majority (55.2%), were less than the age of two years. Both sexes were almost equally represented with the boys-to-girls ratio of 0.98. The majority of children, 354 (92.9%) were newly admitted cases; whereas, 27 (7.1%) were readmitted to the program. Non-edematous acute malnutrition (severe wasting) was the topmost cause of admission (71.4%), followed by edematous acute malnutrition (15.2%) (Table 1).

**Table 1:** Characteristics of children 6 to 59 months of age with SAM admitted to stabilization center at HUCSH, 2018

Characteristics (*n* = 381)	Frequency	Percent
Sex		
Male	189	49.6
Female	192	50.4
Age (months)		
6–11	113	29.7
12–23	97	25.5
24–35	74	19.4
36–47	66	17.3
48–59	31	8.1
Appetite test		
Failed appetite	359	94.2
Passed appetite	22	5.8
Nutritional edema		
Yes	109	28.6
No	272	71.4
Place of residence		
Rural	265	69.6
Urban	116	30.4
Breastfeeding		
Yes	189	49.6
No	192	50.4
Admission status		
New	354	92.9
Readmission	27	7.1
Immunization		
Fully vaccinated	183	48
Partially vaccinated	139	36.5
Unvaccinated	59	15.5
WFH (% of the reference population)		
< 70%	235	61.7
70–79.9%	64	16.8
> 80%	82	21.5
MUAC		
< 11.5 cm	288	75.6
> = 11.5 cm	93	24.4
Admission diagnosis		
Non-edematous malnutrition	272	71.4
Edematous malnutrition	109	28.6

### Medical comorbidity

All children admitted at the stabilizing center had at least one form of co-morbidity. Accordingly, 41.5 and 46.7% of admitted children had pneumonia and diarrhea respectively. The mean (±SD) hemoglobin level (Hb) of admmited children was 9.5 (±2.5) g/dL and 74.5% had anemia (hemoglobin less than 11.0 g/dL). Nearly one-in-five, 22.8% of children were diagnosed with tuberculosis (Table 2).

**Table 2:** Distribution of medical co-morbidities information on treatment outcome of 6 to 59 months of age of children with SAM admitted to stabilization center at HUCSH, 2018

Variables (*n* = 381)	Frequency	Percent (%)
HIV/AIDS		
Positive	3	0.8
Negative	262	68.8
Unknown	116	30.4
Tuberculosis		
Yes	87	22.8
No	294	77.2
Pneumonia		
Yes	158	41.5
No	223	58.5
Fever (body temp > = 37.5 °C)		
Yes	86	22.6
No	295	77.4
Diarrhea		
Yes	178	46.7
No	203	53.3
Type of diarrhea(*n* = 178)		
Watery	173	97.2
Dysentery	5	2.8
Dehydration among children with diarrhea (n = 178)		
Yes	138	77.2
No	40	22.8
Malaria		
Yes	10	2.6
No	339	339
Unknown	32	8.4
Sepsis		
Yes	32	8.4
No	349	91.6
Congestive Heart Failure		
Yes	30	7.9
No	351	92.1
Anemia (Hg level)		
≥ 11.0 g/dl	97	25.5
10.0–10.9 g/dl	77	20.2
7.0–9.9 g/dl	147	38.6
7.0 g/dl	60	15.7

### Routine medication and treatment

Almost all (98.2%) admitted children were received IV antibiotics like Ampicillin and Gentamycin and Ceftrazione. Furthermore, 379(99.5%) of malnourished children received F-75 therapeutic milk while 313(83.2%) and 120(31.5%) received F-100 and plumpyNut (A brand of ready to use theraputic food) respectively. Of the admitted children, 312(81.9%) and 314(82.4%) received Vitamin-A and Folic acid supplementations after admission, respectively. On the otherhand, 27.6% of childen were given deworming medicines (Table 3).

**Table 3:** Routine medication and treatment information of children with SAM admitted to stabilization center at HU-CSH, 2018

Treatment given (*n* = 381)	Frequency	Percent (%)
IV antibiotics		
Yes	374	98.2
No	7	1.8
Blood transfusion		
Yes	29	7.6
No	352	92.4
IV fluid		
Yes	87	22.8
No	294	77.2
Folic acid supplementation		
Yes	314	82.4
No	67	17.6
Vitamin A Supplementation		
Yes	312	81.9
No	69	18.1
F 100 intake		
Yes	317	83.2
No	64	16.8
Plumphy nut intake		
Yes	120	31.5
No	261	68.5
Amoxicillin		
Yes	146	38.3
No	235	61.7
Ampicillin and Gentamycin		
Yes	369	96.9
No	12	3.1
Deworming		
Yes	106	27.8
No	275	72.2
Special medication		
Yes	313	82.2
No	68	17.8
Measles Immunization		
Yes	67	17.6
No	314	82.4

### Treatment outcome

The 381 SAM children admitted at HUCSH stabilization center were retrospectively followed for a variety of periods from a least of 2 days up to a maximum of 59 days. An overall proportion of recovery 272 (69.3%), death 41 (10.8%) and defaulter rate of 27 (7.1%) were observed.

The overall median (IQR) time of recovery was 17(10, 24) days. Nearly half of recovered children were observed during the period of 11–20 days of length of stay. Of all SAM children enrolled in the stabilization center, 41(10.8%) and 19 (7%) died and defaulted, respectively. The average (±SD) weight gain for a severely malnurished children was 12.7 (±8.9) g/kg/day. The mean (± SD) length of stay among recovered edematous and non-edematous children was 10.8 ± 19.0 days and 18.1 (± 11.3) and their difference is not statistically significant (*p* = 0.408). From edematous children 78 (71.6%), 15% (13.8%) and 8 (7.3%) were recovered, died and defaulted respectively. Similarly, out of severely wasted children 186 (68.4%), 26 (9.6%) and 19 (7%) were recovered, died and defaulted respectively.

The overall incidence rate of recovery was calculated using Person-days of follow up as a denominator for the entire cohort. Three hundred eighty one study participants were followed for two years periods which totally gives 6886 Person-days of observation. During the follow up period, 264 children with SAM were recovered. Henceforth, the overall incidence density rate (IDR) of recovery in the cohort was 3.8 per 100 Person-days. The cumulative probability of recovery at the end of one week was 0.97; and recovery at the end of two week was 0.77; that of surviving at the end of four week was 0.29 and that of surviving at the end of eight week was 0.07(Table 4).

**Table 4:** Life table analysis of severely acutely malnourished children treated at HUCSH stabilization center Southern Ethiopia from July 2015 to June 2017

Interval Start Time (days)	Number Entering Interval	Number Withdrawing during Interval	Number Exposed to Risk	Number of Terminal Events	Proportion Terminating	Proportion Surviving	Cumulative Proportion Surviving at End of Interval
0–7	381	34	364.000	10	.03	.97	.97
7–14	337	38	318.000	67	.21	.79	.77
14–21	232	12	226.000	85	.38	.62	.48
21–28	135	10	130.000	51	.39	.61	.29
28–35	74	5	71.500	33	.46	.54	.16
35–42	36	5	33.500	16	.48	.52	.08
42–49	15	6	12.000	0	.00	1.00	.08
49–56	9	5	6.500	1	.15	.85	.07
56–63	3	2	2.000	1	.50	.50	.03

### Factors associated with recovery time of severely malnourished children

Bivariable Cox regression analysis was run for the following independent variables, Sex, Age, place of residence, Immunization status, breastfeeding, appetite test, WFH, MUAC, edema, type of Diagnosis, comorbidities and routine medication provision. Subsequently, the bivariable analysis finding showed that Tuberculosis (CHR = 1.38, 95% CI = 1.02–1.87), Malaria(CHR = 2.48, 95% CI = 1.02–6.02), special medication provision(CHR = 1.61, 95% CI = 1.19–2.18), F-100 intake (CHR = 2.09, 95% CI = 1.23–3.57), and deworming (CHR = 1.41, 95% CI = 1.08–1.85) were found to be significant predictors to time-to-recovery. In addition, pneumonia (CHR = 1.15, 95% CI = 0.90–1.48), Septic shock (CHR = 3.20, 95% CI = 0.40–22.70), breastfeeding at the time fo the survey (CHR = 0.80, 95% CI = 0.6–1.0), and Fever (CHR = 0.82, 95% CI = 0.61–1.11) were found to have *p* value of < 0.25, hence considered eligible for the multivariable analysis (Table 5).

**Table 5:** Output of Bivariable and Multivariable Cox regression analyses on factors associated with time-to-recovery of 6 to 59 months of age of children with SAM admitted to stabilization center at HUCSH, 2018

Variables	Outcome		CHR (95% CI)	AHR (95% CI)
	Recovered	Censored		
Age				
≤ 24 months	193 (73.1)	85 (72.6)	1	1
> 24 months	71 (26.9)	32 (27.4)	0.89 (0.68–1.18)	0.93 (0.71–1.21)
Appetite test				
Yes	13 (4.9)	9 (7.7)	1	1
No	251 (95.1)	108 (92.3)	0.59 (0.34–1.04)	0.66 (0.36–1.20)
Tuberculosis				
Yes	53 (20.1)	34 (29.1)	1	1
No	211 (79.9)	83 (70.9)	1.38 (1.02–1.87)	1.38 (1.00–1.91)*
Pneumonia				
Yes	105 (39.8)	53 (45.3)	1	1
No	159 (60.2)	64 (54.7)	1.15 (0.90–1.48)	1.15 (0.89–1.49)
Malaria				
Yes	5 (1.9)	5 (4.3)	1	1
No	237 (89.8)	102 (87.2)	2.48 (1.02–6.02)	2.11 (0.86–5.15)
Unknown	22 (8.3)	10 (8.5)	2.11 (0.80–5.59)	1.92 (0.72–5.16)
F 100 intake				
Yes	249 (94.3)	68 (58.1)	1	1
No	15 (5.7)	49 (41.9)	2.09 (1.23–3.57)	0.50 (0.29–0.86)*
Body temp.				
≥ 37.5 °C	53 (20.1)	33 (28.2)	1	1
< 35.5 °C	211 (79.9)	84 (71.8)	0.82 (0.60–1.11)	1.20 (0.88–1.64)
Deworming				
Yes	77 (29.2)	29 (24.8)	1	1
No	187 (70.8)	88 (75.2)	1.41 (1.08–1.85)	0.78 (0.58–1.05)
Special Medication				
Yes	210 (79.5)	103 (88.0)	1	1
No	54 (20.5)	14 (12.0)	1.61 (1.19–2.18)	0.72 (0.52–0.99)*

*show statistically significant association between predictors and time to recovery (*P* <  0.05)

Multivariable Cox regression was carried out for variables verified as significant at *p* = value, < 0.25 by bivariate Cox regression. Accordingly, after adjusting for different variables F-100 intake, Tuberculosis infection and provision of special medication at admission were found to be independent predictors of recovery time in severely malnourished children admitted to HU-CSH.

Accordingly, severely malnourished children who were not recieved F-100 therapeutic milk had a 50% reduced recovery time than those children who recieved F-100 therapeutic milk (AHR = 0.50, 95%, CI: 0.29–0.86). Children who were admitted with out Tuberculosis infection were 1.4 times more likely of recovery as compared to those who didn’t have Tuberculosis as a comorbidity (AHR = 1.38, 95% CI: 1.00–1.91). Likewise, children who were not treated with a special type of medication had a 28% reduced chance of nutritional recovery as compared to those who were treated with it (AHR = 0.72, 95% CI: 0.52–0.99) (Table 6).

**Table 6:** Log rank (Mantel-Cox) test for association of explanatory variables with time to recovery from complicated SAM children admitted at HU-CSH from July 2015 to July 2017

Variables	Mean recovery time(95% CI)	Over all comparison Log Rank	
		*X* ^*2*^	*P-*value
Received special medication			
Yes	18.1 (15.8–20.4)	10.429	0.001
No	24.2 (22.4–25.9)		
Tuberculosis			
Yes	21 (16.2–25.7)	4.795	0.029
No	20 (18.8–21-1)		
F-100 therapeutic food intake			
Yes	23.5 (22.0–25.1)	8.125	0.004
No	18.6 (14.1–23.0)		
Type of malnutrition			
Non-edematous	23.1 (21.3–25.0)	0.001	0.970
Edematous	23.2 (20.4–25.9)		

Time-to-recovery patterns of the SAM children across selected variables were compared using Log-rank test. Hence, there were significantly different recovery rates among children with and without Tuberculosis infection. The mean recovery time with the presence and absence of Tuberculosis infection in complicated SAM children was 26.1 and 22.0 days respectively and their difference was statistically significant (Log-rank test = 4.79, *P* = 0.029) (Table 6). Similarly, children who received and not received F-100 diet (Log rank test = 8.12,*P* = 0.004) and children who managed with and without special medication (Log-rank test = 10.43, *P* = 0.001) were fund to be a significant factors affecting recovery. However, there was no difference in the recovery times between the types of malnutrition (Log rank = 0.751, *P* = 0.687) (Table 6).

## Discussion

The finding of this study revealed that the overall recovery from complicated SAM after a maximum of 59 days treatment was (69.4%) and 10.8% of children end up in death. The overall incidence density rate (IDR) of recovery in the cohort was 3.8 per 100 Person-days. The presence of Tb infection, giving F-100 therapeutic milk and provision of special medication were identified as significant predictors of time to nutritional recovery.

The finding of this study revealed that a recovery rate of 69.4%, which is below the minimum SPHERE standard,which states recovery rate should be greater than or equal to 75% [13]. The result of this study is in line with similar other studies conducted in Ethiopia: Sekota [6], Debere Brehan Referal Hospital [14], and Bahirdar town [15]. Studies conducted else where also concluded the same Zambia and India [16]. However, the finding of this study was inconsistent with the similar study conducted in Ethiopia: Woldia [17], Jimma [18], Debre markos and Finote Selam Hospitals [19].

This implies a recovery rate from complicated SAM in these children who had been managed at HU-CSH stabilization center is unacceptably low. The most likely reasons mentioned for the low recovery rate might beincreased patient flow to the study hospital from different parts of the region through referral with presence of high burden of comorbidities such as Tb, Malaria and severe anemia and increament of death rate, transfer out and medical transfer in this study could be responsible for the observed low recovery rate.

The finding of this study revealed that, HUCSH stabilization center had death rate greater than the minimum standard indicated for inpatient management of SAM. Other studies also reported death rate ranging from 15.2 to 29% [6, 12]. Nearly 60% of deaths were occurred within the first week after initiating treatment, so this might be related to stabilization center factors such as excess case-load and shortage of trained staff. In this study facility merely three nurses were trained on the management of complicated SAM despite the high annual patient flowin the pediatrics department.

The study revealed that, children who defaulted (7.1%) was considerably below the national and international minimum standards of cut-off point (< 15%) [2, 13]. A similar finding ranging from 5.3 to 12.9% was also obtained in different studies conducted in Ethiopia [6, 12, 14, 19, 20]. The average length of stay under the stabilization center was 18.3 days. This is in line with the acceptable minimum international standard [13] and Ethiopian protocol for management of SAM which recommends children to stay under treatment less than 28 days [2].

Correspondingly, the length of stay in this study is consistent with other analogous studies conducted in Jimma University Specialized Hospital (17.4 days) and Bahirdar Felege Hiwot Hospita (18.0 days) [15, 20]. However, the finding in this study is incomparable to similar studies conducted in Gedeo Zone Hospitals (13 days), Sekota Hospital Waghemra Zone (9 days) [7], Woldia Hospital (14 days) [18] and India (13.2 days) [17]. The disparities among the length of stay might be due to settings difference, severity of illness at basline, case overload at the health instituions, and availability of skilled and trained staffs or as a result of the presence of comorbidities.

The study revealed that, the average weight gain of SAM children under the HUCSH stabilization center was 11.2 g/kg/day (SD ± 8.6), which is satisfactorily comparable with international and national standard cutoff point of minimum average weight gain of ≥8 g/kg body weight/day [2, 13]. This finding was consistent with similar study conducted in India (12.1 g/kg/d), Woldia (12.0 g/kg/d), Jimma (10.4 g/kg/day) and Wolaita (15.3 g/kg /d) [16, 17, 20, 21]. Conversely, the finding is by far greater than similar study conducted in Gedeo Zone (8.7 g/kg/day) [12]. The result may be due to proper management of SAM and provision of therapeutic diet and routine medication and staying less than the recommended period of time in the program.

Both the rate of weight gain and the average length of stay in the program is within optimal range. However, the observed recovery rate is lower as compard to the national and international SPHERE standared. The low recovery rate might be atrributed by high death rate 10.8%, defaulter rate 7.1% and medical transfer 8.1%. likewise, this might diminishes the sample size for recovered children. Additonally, the shortest in average length of stay in the programme is also be a factor for increament of the average weight gain.

Out of total admissions to the HUCSH stabilization center OTP, 7.1% were readmitted cases. This number of readmission cases may be due to lack of daily follow at the OTP site, poor linkage of health facilities and absconded from OTP therapeutic program. A similar finding ranging from 4.4 to 22% was also obtained in different studies conducted in Ethiopia [19, 21, 22].

Tuberculosis prevalence, 22.8% found among children in this study is by far higher than similar study conducted in Sekota Hospital 2.2% [6]. Zambia Lusaka Hospital 5.3% [23], Bahir Dar Felege Hiwot Hospital 9.2% [15] and Wolaita Hospital [20]. Accordingly, SAM children admitted at HUCSH stabilization center without Tb infection had 1.4 times more likely recovery time (AHR: 1.38) than children with Tb infection. This is comparable with similar study conducted in Sekota Hospital [6] and Bahirdar Felege Hiwot Hospital [15]. However, the finding of this study is inconsistent with a study that had been conducted in Zambia lusaka [23], Jimma [20], and Woldia [17]. The possible reasons behind might be because the study area is a tertiary hospital.

Moreover this study revealed that children who did not received F-100 therapeutic milk had 50% reduced recovery time than who took F-100 therapeutic milk (AHR: 0.50). This is the fact that F-100 has higher calorie 100 kcal/100 ml, which increases the daily weight gain and improves the outcome of a child [1].

On the other hand, children not received special medications at admission had 28% reduced recovery time than those children who had received special medication (IV fluid, IV antibiotics and blood transfusion) (AHR = 0.72). However, this is not comparable with similar study conducted in Jimma University Specialized Hospital [20]. This might be due to the difference in the type of SAM admission and comorbidities. Further, it implies the study hospital had an appropriate nutritional therapy in combination with the national SAM management protocol promote early recovery from the program.

The Strengths of the Study was using of advanced statistcal analysis model. Further, this is study is among few studies in Ethiopia, that were conducted to assess treatment outcome of and factors affecting time to recovery among 6–59 months old children with SAM admitted at HUCSH stabilization center. The limitationsof the study was lack of complete data and consistence, poor archive of old registration books, missing to record some portion of charts by ward log books compromise the sample size. Becaues the study was relied on secondary data analysis of all risk factors associated with time to recovery was restricted. In addition to incomplete nature of secondary data.

## Conclusion

The finding of this study revealed that the overall recovery from complicated SAM after a maximum of 59 days treatment was low (69.4%) and a very high proportion of children (10.8%) end up in death.

The overall incidence density rate (IDR) of recovery in the cohort was 3.8 per 100 Person-days. The median time of recovery was 17 (IQR 10–24) days. Tuberculosis infection, F-100 therapeutic diet provision and prescription of special medication were the variables which are significantly associated with recovery time among the severely malnourished children managed at HUCSH stabilization center. HUCSH Pediatrics department should give special focus for those who present with medical comorbidities during inpatient stabilization center. The health care providers have to be strongly advised to comply with WHO recommended complicated SAM treatment protocol. For additional enquiry, we suggest a prospective cohort study on problems related to nonresponse to treatment and readmission.

## Abbreviations

AHR: Adjusted hazard ratio
CHR: Crude hazard ratio
CI: Confidence interval
CSA: Central statistical agency
CTC: Community based therapeutic center
EDHS: Ethiopian demographic and health statistic
FMOH: Federal ministry of health
HIV: Human immune deficiency virus
MUAC: Mid upper arm circumference
RUTF: Ready-to-Use therapeutic food
SAM: Severe acute malnutrition
SC: Stabilization center
SPHERE: State wide partnership for HIV education in recovery environments
UNICEF: United nation international children’s fund
WFH: Weight for height
WFP: World food program
WHO: World health organization

## References

[1] WHO (2013). Guideline: Updates on the management of severe acute malnutrition in infants and children.

[2] Federal Ministry of Health [Ethiopia]. Protocol for the Management of Acute Malnutrition. Addis Ababa MoH; 2007. Google Scholar.

[3] Black RE, Allen LH, Bhutta ZA, Caulfield LE, de Onis M, Ezzati M (2008). Maternal and child undernutrition: global and regional exposures and health consequences. Lancet.

[4] Central Statistical Agency (CSA) [Ethiopia] and ICF (2016). Ethiopia Demographic and Health Survey 2016: Key Indicators Report.

[5] UNICEF Program E Guidance Document. Management of Severe Acute Malnutrition in Children: Working Towards Results Scale. New York, NY 10017, USA, United Nations Children’s Fund; 2015. http://www.childrenandaids.org/sites/default/files/2017-05/SAM%20Guidance.pdf. Accessed 14 Jan 2018

[6] Shitaye DK (2015). Survival Status and Predictors of Mortality among Children Aged 0–59 Months with Severe Acute Malnutrition Admitted to Stabilization Center at Sekota Hospital Waghemra Zone. Nutr Disord Ther.

[7] Shanka NA, Lemma S, Abyu DM. Recovery rate and determinants in treatment of children with severe acute malnutrition using outpatient therapeutic feeding program in Kamba District, south West Ethiopia. Nutr Disord Ther. 2015;5(2). 10.4172/2161-0509.1000155. ViewArticleGoogle scholar.

[8] UNICEF (2012). Evaluation of Community Management of Acute Malnutrition (CMAM): Ethiopia Country Case Study.

[9] Collins S, Dent N, Binns P, Bahwere P, Sadler K, Hallam A (2006). Management of severe acute malnutrition in children. Lancet.

[10] Global Nutrition Report. Actions and accountability to accelerate the World’s Progress on nutrition. Washington, DC USA: 2014This report is found the link (URL): http://globalnutritionreport.org/documents/5/128695.pdf. Accesed 14 Jan 2018.

[11] Ethiopia Federal Ministry of Health. Revised Health Management Information System Indicator Definition. March, 2014. https://www.measureevaluation.org/our-work/routine-health-information-systems/rhis-curriculum-modules/handout-2-2.4b. Accesed 14 Jan 2018.

[12] Moges T, Haidar J (2009). Management and outcome of severely malnourished children admitted to Zewditu memorial hospital, Ethiopia. East Afr J Public Health.

[13] The SPHERE Project: Humanitarian Charter and Minimum Standards in disaster response; 2004. Google Scholar. 10.1111/j.0361-3666.2004.00245.x 20958782

[14] Derseh B, Mruts K, Demie T, Gebremariam T. Co-morbidity, treatment outcomes and factors affecting the recovery rate of under -five children with severe acute malnutrition admitted in selected hospitals from Ethiopia: retrospective follow up study. Nutrition Journal. 2018;17(116). 10.1186/s12937-018-0423-1. 10.1186/s12937-018-0423-1 PMC6299567 30563516

[15] Desyibelew HD, Fekadu A, Woldie H (2017). Recovery rate and associated factors of children age 6 to 59 months admitted with severe acute malnutrition at inpatient unit of Bahir Dar Felege Hiwot Referral hospital therapeutic feeding unite, northwest Ethiopia. PLoS ONE.

[16] Singh K, Badgaiyan N, Ranjan A, Dixit H, Kaushik A (2014). Management of children with severe acute malnutrition: experience of nutrition rehabilitation centers in Uttar Pradesh, India. Indian Pediatr.

[17] Chane T, Oljira L, Atomesa GE, Agedew E (2014). Treatment outcome and associated factors among under-five children with severe acute malnutrition admitted to therapeutic feeding unit in Woldia hospital, North Ethiopia. J Nutr Food Sci.

[18] Jarso H, Workicho A, Alemseged F (2015). Survival status and predictors of mortality in severely malnourished children admitted to Jimma University specialized hospital from 2010 to 2012, Jimma, Ethiopia. BMC Pediatrics.

[19] Mekuria G, Derese T, Hailu G (2017). Treatment outcome and associated factors of severe acute malnutrition among 6–59 months old children in Debre Markos and Finote Selam hospitals, Northwest Ethiopia. BMC Nutrition.

[20] Girum T, Kote M, Tariku B, Bekele H (2017). Survival status and predictors of mortality amongN severely acute malnourished children <5 years of age admitted to stabilization centers in Gedeo zone. Terapeutics Clin Risk Manag.

[21] Nyeko R, Calbi V, Ssegujja BO, Ayot GF (2016). Treatment outcome among children under- five years hospitalized with severe acute malnutrition in St. Mary’s hospital Lacor, northern Uganda. BMC Nutrition.

[22] Mengesha MM, Deyessa N, Tegegne BS, Dessie Y (2016). Treatment outcome and factors affecting time to recovery in children with severe acute malnutrition treated at outpatient therapeutic care program. Glob Health Action.

[23] Munthali T, Jacobs C, Sitali L, Dambe R, Michelo C (2015). Mortality and morbidity patterns in under-five children with severe acute malnutrition (SAM) in Zambia: a five-year retrospective review of hospital-based records (2009–2013). Arch Public Health.

